# Changes in the Microbiome During Chronic Rhinosinusitis

**DOI:** 10.3390/pathogens14010014

**Published:** 2024-12-30

**Authors:** Mateusz de Mezer, Nina Chalama, Cheyanna Bratt, Melanie Kiebalo, Natalia Dolata, Jan Rogaliński, Małgorzata Leszczyńska

**Affiliations:** 1 Department of Immunobiology, Poznań University of Medical Sciences, Rokietnicka 8 St., 60-812 Poznań, Polandjrogalinski@ump.edu.pl (J.R.); 2 Department of Otolaryngology and Laryngological Oncology, Poznań University of Medical Sciences, Przybyszewskiego 49 St., 60-355 Poznań, Poland; chalamanina@gmail.com (N.C.); cbratt1992@gmail.com (C.B.); mkiebalo@gmail.com (M.K.)

**Keywords:** chronic rhinosinusitis (CRS), nasal polyps, microbiome, dysbiosis, inverted papilloma, upper airway, otolaryngology

## Abstract

Chronic rhinosinusitis (CRS) is a common inflammatory disease of the paranasal sinuses with a yet unknown etiology. As studies continue to elucidate the disease’s heterogeneity inflammatory profile and presentation, there is a growing interest in the influence of the nasal microbiome on disease pathogenesis and chronicity. The sinus microbiota appear dominated by the *Staphylococcus* and *Corynebacterium* genera; known upper airway pathogens, such as *Haemophilus influenza*, are present in the upper airways of healthy individuals, though at relatively lower abundances than in CRS patients. Viral culprits may induce an unhindered local immune response that contributes to the recurrence and chronicity of inverted papillomas—benign mucosal lesions with the propensity for local destruction and malignant transformation that can be found in patients with a history of nasal infection. The persistence of inverted papillomas warrants investigation into their pathogenesis and how they may contribute to a nasal landscape promoting the chronicity of CRS. Further investigation is needed to uncover the interplay between resident microbiota and viral, fungal, and immunological influence. Discerning between ‘healthy’ and ‘diseased’ sinonasal microbiomes and ‘keystone’ species could shed light on CRS etiology and provide the opportunity for CRS treatment tailored to an individual’s microbiome. This review aims to explore the interrelation of microbial residents in the pathogenesis and chronicity of the diseased sinonasal environment.

## 1. Introduction

CRS in adults is defined as the presence of two or more symptoms:

- one of which is either nasal blockage, obstruction, congestion, or nasal discharge;
- +/− facial pressure/pain;
- +/− reduction or loss of smell

For over 12 weeks. Patients bear a high symptom burden that compromises mental, emotional and physical function [1]. CRS patients report a greater impairment of quality of life than patients with other chronic diseases, such as congestive heart failure and angina [2]. The prevalence of recalcitrant patients—those for whom symptoms persist despite long-term medical and surgical intervention—highlights the limitations of contemporary treatments and the need for individualized therapy grounded on a clearer understanding of the disease’s yet unknown etiology.

CRS has been traditionally classified into two groups based on the presence or absence of polyps: CRS with nasal polyps (CRSwNP) or CRS without nasal polyps (CRSsNP). These phenotypes are limited in describing the full spectrum of CRS disease variants. The European Position Paper of Sinusitis and Nasal Polyps 2020 (EPOS 2020) provides a classification of CRS into primary and secondary CRS, with further division based on anatomic distribution [3].

The EPOS 2020 first divides the primary CRS subtype by anatomic distribution: inflammation and/or polyps localized unilaterally or diffusely bilateral. The disease is then classified by endotype dominance: a type 2 or non-type 2 inflammation pattern.

The non-type 2 categorization belies the heterogeneity of the disease. Until recently, CRSwNP was thought to present with a type 2 inflammatory profile, but studies continue to elaborate on associations between inflammation types and geographical distribution. While American and European CRSwNP patients largely demonstrate a type 2 response, Asian CRSwNP populations trend towards neutrophil-dominant inflammation [4].

**Figure 1:**
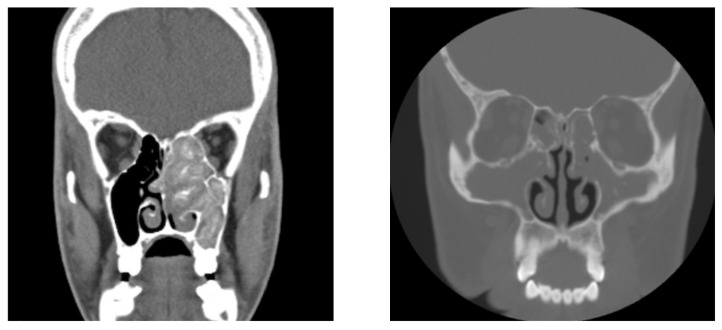
(**Left**) Coronal CT of allergic fungal rhinosinusitis, with opacification and expansion of paranasal sinuses. (**Right**) Coronal CT scan of paranasal sinuses with features consistent with chronic rhinosinusitis, with opacification and possible bony erosion consistent with invasive infections. Images courtesy of the Otolaryngological and Radiological Departments of Heliodor Święcicki Hospital.

Localized CRS is categorized by clinical phenotype: allergic fungal rhinosinusitis (AFRS) (Figure 1) or isolated sinusitis. Diffuse CRS is predominantly subdivided into eosinophilic CRS (eCRS) or non-eCRS, determined by an eosinophil count of 10 per high power field or higher [3].

Aspirin exacerbated respiratory disease (AERD), also known as NSAID exacerbated respiratory disease (NERD) and Samter’s Triad, is a triad of CRSwNP, asthma, and intolerance to cyclooxygenase 1 (COX 1) inhibitors, such as aspirin. AERD is a chronic eosinophilic inflammatory disorder of the airways in which symptoms are exacerbated by the use of NSAIDs. NERD pathogenesis is related to irregular eicosanoid synthesis, leading to eosinophilic inflammation of nasal and sinus membranes and increased leukotriene production [3]. AERD patients undergo twice as many surgeries than non-AERD CRSwNP patients, and are significantly younger during their first surgery.

CRSwNP patients have a high prevalence of comorbid asthma, with one study reporting an asthma prevalence among CRSwNP patients at a tertiary allergy center in Kuwait to be 59.63% [5]. CRS patients with more severe respiratory disease are more likely to undergo surgical treatment for polyps, which may in turn exacerbate the respiratory microenvironment [6]. Correctly discerning CRSwNP with or without comorbid asthma from AERD is critical to prevent the delay of appropriate treatment and avoid further confounding of either disorder’s clinical course.

Via EQ-5D and SNOT-2 questionnaires, Wahid et al. determined that CRS patients in the UK reported significantly more primary care and secondary care (that is, care sought after initial medical treatment failed) than non-CRS controls over a 3-month duration: 4.14 vs. 1.16 for primary care visits, and 2.61 vs. 0.40 for secondary care visits. Individuals with CRS reported missing an average of 18.7 workdays per year due to poor health and symptomatic burden. With the estimated healthcare cost of CRS totaling GBP 16.8 billion per year, the disease impacts the economy beyond immediate healthcare costs [7].

The bulk of the literature detailing the socioeconomic impact of CRS is limited to the United States, where the direct cost of CRS is estimated at US 10–13 billion per year. Disease exacerbation results in absenteeism, lost wages, and lost productivity—these indirect costs are estimated to exceed $20 billion annually [8,9].

In a meta-analysis of 45 studies with 34,220 patients, Loftus et al. propose that the large surgical revision rate in CRSwNP patients is driven by certain “high-risk” groups: patients with coexisting aspirin exacerbated respiratory disease (AERD), allergic fungal rhinosinusitis (AFRS), or cystic fibrosis. Also identified were higher revision rates in North America and Oceania, versus Europe and Asia. This difference is proposed to relate to underlying inflammatory patterns; in Asian countries, CRSwNP is non-eosinophil dominant with higher rates of neutrophilic CRSwNP profiles. In contrast, CRSwNP tissues from Western countries show a higher proportion of eosinophilic-predominant disease [10].

## 2. Methodology of Microbiota Sampling

Analysis of the microbial community is feasible as samples are taken from patients at the onset of endoscopic surgery. The middle meatus is proposed as a representative sampling site for the deeper sinuses, due to similar cultural comparison to the maxillary sinus, its location as a common drainage pathway for the maxillary, anterior ethmoid, and frontal sinuses, and its accessibility for sampling [11,12]. The high interpersonal variation of microbial composition among sampled patients outweighs heterogeneity that may arise from swab samples collected from different anatomical sites [13]. Tissue specimens may be more inclusive in biomass collection than swabs, as they are better able to collect biofilms that grow on and penetrate mucosal epithelium [14]. Bacterial profiles are often rendered by 16S rRNA sequencing, though the measure of the abundance of bacterial DNA does not distinguish between living or dead biomass [11]. Interactions between select species can be further observed by culture. During the COVID-19 pandemic, various diagnostic methods were adapted to new purposes. This has enabled the differentiation of species of nasal sinus pathogens by using mass spectrometry and its variant MALDI-TOF [15,16] as well as electron microscopy [17]. These methods are fast and allow for precise analysis of material from nasal swabs, so they do not require the collection of larger tissue fragments by surgery.

Though viral and fungal presence in CRS has been demonstrated, there is a lack of investigation into how nasal consortia interact with non-bacterial pathogens [11].

## 3. The Healthy Microbiome and the Dysbiosis Hypothesis

We now know that a healthy sinus is neither sterile nor necessarily stable. Interpersonal microbial heterogeneity may be due to temporal fluctuations of community composition; consequences of long-term steroid and antibiotic use in the CRS population have yet to be determined by longitudinal study.

The sinonasal microbiome seems to consist of core organisms that distinctly co-occur, such as *Staphylococcus aureus* and *Corynebacterium* sp., which may have a reciprocal or antagonistic relationship [18]. Commonly identified bacteria from the upper airways in the healthy state include *Staphylococcus*, *Corynebacterium*, and *Propionibacterium* [13,19].

The prevalence of inter-species interaction led Bassiouni et al. to propose categorizing the sinonasal microenvironment (irrespective of healthy or CRS states) into one of three ‘microbiotypes’: *Corynebacterium*-dominated, *Staphylococcus*-dominated, and the third, a heterogenous microbiotype dominated by other core genera of the sinonasal microbiome (*Streptococcus*, *Haemophilus*, *Moraxella*, and *Pseudomonas*) [18].

Characteristics of the most common species in sinonasal-microbiome *Staphylococcus* spp. are Gram-positive coccal-shaped, facultative anaerobic bacteria, members of the *Staphylococcacae* family. They are one of the most abundant skin-colonizing bacteria [20,21]. Among these genera the most popular bacteria are *S. aureus* and *S. epidermidis*, but the other species, such as *S. haemolyticus*, *S. saprophyticus*, *S. hominis*, etc., could be named as “unclassified Staphylococcus” [18]. Even though *Staphylococcus aureus* is one of the most common pathogens in skin and soft tissue infections [22], it colonizes nares in around 30% of the human population [23]. However, the methicillin-resistant *Staphylococcus aureus* (MRSA) maintains a significant role in chronic rhinosinusitis due to its antibiotic resistance [24,25] and it could be found in even 60% of *S. aureus* isolates from sinonasal swabs [26].

*Corynebacterium* spp. are Gram-positive, rod-shaped, aerobic, and facultative anaerobic bacteria, members of the *Corynebacteriaceae* family and, like *Staphylococci*, are part of the physiological skin microbiome [21]. Through its genera, we can distinguish those with the highest clinical value: *C. diphteriae*, *C. ulcerans*, and *C. jeikeium* [27,28,29]. *Corynebacterium* spp. and *Staphylococcus* spp. were the most abundant genera of sinonasal microbiome in healthy controls according to studies of Roh et al. [27].

*Propionibacterium* spp. are Gram-positive, anaerobic bacteria, members of the *Propionibacteriaceae* family, and includes species like: *P. acnes*, *P. avidum*, and *P. granulosum*. *P. acnes* (renamed *Cutibacterium acnes*) is present in skin pores, where it dominates the bacterial flora [30]. It is strictly associated with acne, and has become a subject of multiple studies, also about managing this disease [30,31,32,33].

## 4. Bacterial Interactions

Roh et al. presented that microbiome composition differs between CRS and healthy patients [27]. In healthy controls *Staphylococcus* spp. were the most enriched (56.9%), followed by *Corynebacterium* spp. (16.8%) and *Achromobacter* spp. (10.6%). CRS patients also have a sinonasal microbiome dominated by *Staphylococcus* spp. (39.7%), but the other most abundant were: *Lactobacillus* spp. (9.7%) and *Corynebacterium* (5.9%), which is much lower than in healthy controls [27].

There is a hypothesis that *Corynebacterium* spp. protects from pneumococcal colonization in children due to its antagonistic relationship [28,29]. Some reports have presented a theory about the commensal-pathogen relationship between *Corynebacterium* spp. and *Staphylococcus aureus*, though authors have presented different results [28]. For example, Wos-Oxley et al. show that *S. aureus* is negatively correlated with *C. accolens* but positively correlated with the *C. pseudodiphtheriticum* genus in healthy adults [34]. On the other hand, in patients with persistent *S. aureus* colonization, Yan et al. found positive correlation with *C. accolens* and more abundant growth [35]. These authors also show that *C. pseudodiphtheriticum* is present in *S. aureus* non-carriers more often than expected, which suggests competitive interactions, in contrast to the studies mentioned above.

Another commensal-pathogen relationship is observed between *P. acnes* (*C. acnes*) and *S. aureus*, in which a denser biomass biofilm is formed while in coexistence; however, the presence of *P. acnes* supernatants increases the antibiotic susceptibility of *S. aureus* [36,37]. Other studies on reference strains show that propionic acid produced by *P. acnes* inhibits the growth of *S. aureus* USA300 CA-MRSA both in vitro and in mice wounds [38]. On the other hand, the *P. acnes*’ CAMP factor enhances virulence factors such as the haemolytic and cytolytic capabilities of *S. aureus* [39]. Although some of these studies were conducted on skin-related models, it is likely that they are comparable to interactions within nasal and sinonasal passages [28].

All these reports suggest that enhancement or inhibition could not be the only interactions between microorganisms in sinonasal cavity, and some molecular mechanisms can be involved.

## 5. The Impact of the Microbiota on CRS: Diversity, Dysbiosis, and Recurrence Profile

Examining the microbiome of healthy sinuses, Ramakrishnan et al. analyzed middle meatal swabs from 28 patients without sinusitis undergoing endoscopic surgery. Taxa of a healthy middle meatus were collected by broad-range PCR and pyrosequencing of bacteria 16S rRNA gene sequences. *Staphylococcus epidermidis*, *Staphylococcus aureus*, and *Propionibacterium acnes* were the three most prevalent and relatively abundant species found. *Corynebacterium tuberculostearicum* was the dominant *Corynebacterium* species; *Corynebacterium* species were collectively prevalent in 26 out of 28 patients. Ramakrishnan et al. remark that these prevalences reflect prior analysis of the anterior nares in healthy subjects. The middle meatus yielded known or potential opportunistic pathogens at low relative abundances: *Streptococcus pneumoniae*, *Neisseria meningiditis*, *Haemophilus influenzae*, and *Moraxella catarrhalis* [13]. Heterogeneity of relative abundances in even the most prevalent bacteria was found on a person-to-person level.

Chalermwatanachai et al. identified significant heterogeneity in the sinonasal bacterial community, even among persons of different CRSwNP endotypes. Endoscopically guided middle meatus swabs from asthmatic CRSwNP patients (CRSwNP+A), CRSwNP patients without asthma (CRSwNP-A), and healthy, non-sinusitis controls were compared [40]. Control subjects and patients CRSwNP had the same total bacterial load, but CRSwNP patients, (irrelevant of comorbid asthma), showed significantly decreased diversity compared to the healthy control. *Haemophilus influenzae* was present in higher abundance in CRSwNP patients compared to healthy subjects. CRSwNP-A patients had a higher abundance of *Corynebacterium* and *Geobacter* compared to CRSwNP-A patients. *Moraxella catarrhalis*, *Staphylococcus aureus*, and *Staphylococcus xylosus* were less prevalent in CRSwNP-A patients compared to the other groups. *Propionibactetrium acnes*, a commensal of human skin, was the most abundant species of the healthy cohort [40].

The ‘dysbiosis hypothesis’ proposes that inflammatory diseases are associated with significant shifts in the resident microbiota from a ‘healthy’ to a ‘diseased’ state. Antibiotics and steroids are the mainstays of treatment for CRS. CRSwNP patients undergo FESS to remove polyps, with ‘functional’ referring to the restoration of nasal physiology. Postoperative changes to the nasal landscape may alter its microbiota into a ‘diseased’ state of chronic inflammation. Microbial dysbiosis is increasingly thought to be due to the altered balance of bacterial relationships, rather than driven by a single pathogen.

Certain microbial profiles could predict disease recurrence and allow for early treatment. Zhao et al. investigated whether nasal microbiota correlated to CRSwNP patients with recurrence of polyps [41]. Despite subject heterogeneity, the abundance of *Proteobacteria* and *Firmicutes* genera distinguished significantly between the two groups; *Campylobacter*, *Bdellovibrio*, and *Aggregatibacter* were higher in patients with recurrence, while *Actinobacillus*, *Gemella*, and *Moraxella* showed to be more prevalent in the non-recurrence group. Shewanella and Peptostreptococcus were markedly decreased in the non-recurrence group, while Friedmanniella, Curvibacter, and Pseudoxanthomonas were more abundant in the recurrence group. Using the LASSO regression model, *Porphyromonas*, *Bacteroides*, *Moryella*, *Aggregatibacter*, *Butyrivibrio*, *Shewanella*, *Pseudoxanthomonas*, *Friedmanniella*, *Limnobacter*, and *Curvibacter* were identified as taxa most predictive of polyp recurrence [41].

Little consideration has still been given to the extra-sinonasal microbiological impact on sinusitis chronicity. CRS treatment may disturb the intestinal microbiome; in turn, the gut microbiota imbalance is hypothesized to influence a chronic disease state. Michalik et al. found significant alterations in the gut microbiota of CRS patients and CRS patients with non-sinusitis comorbidities (e.g., flatulence and autism). All CRS patients were found to have altered gut microbial composition, compared to standard reference values [42]. Of note, CRS patients had lower amounts of *Bifidobacterium*, *Akkermansia muciniphila*, and *Faecalibacterum prausnitzii*–species suggested to benefit health. *Bifidobacterium* spp., for instance, is one of the most abundant gut bacteria known to produce vitamin B, folic acid, and restore microbiological balance after antibiotic treatment [42].

## 6. Biofilms in CRS

A biofilm is a complex community of microbes embedded within a self-produced, surface-adherent extracellular matrix. The adaptability and evasiveness of these microbial collectives make biofilms a leading cause of chronic infections; they are difficult to eradicate with antimicrobial treatment since they are hotspots for the exchange of genetic material that can rapidly confer antimicrobial resistance, potentially contributing to disease recalcitrance [43].

Długaszewska and colleagues analyzed nasal concha mucosa specimens from 30 CRS patients taken during FESS and from 20 non-sinusitis, non-polyped control patients taken during nasal septoplasty and rhinoplasty [44]. Biofilm was detected by scanning electron microscopy (SEM). Biofilm was consistently present in CRS samples, as was marked destruction of epithelium, ranging from disarrayed cilia to the total absence of cilia. A total of 23 of 30 patients undergoing FESS for CRS had evidence of biofilms on SEM micrographs. Of the 62 microorganisms isolated from 29 CRS samples, *Staphylococcus epidermidis* was the most frequently found, and other coagulase-negative *Staphylococcus* were present in 86.6% of samples; *S. aureus* was present in 23% of samples. The assessment of isolated aerobic bacteria from CRS patients found that most were considered weak or moderate biofilm producers, while five isolates were considered strong biofilm producers (*E. coli*—2 strains; *S. epidermidis*—2 strains; *C. freundii*). As biofilm formation was also seen in 45% of control patients, a cohort without evidence of chronic inflammation, the role of biofilm in CRS etiology should be re-evaluated [44].

## 7. Viral Infection as a Predisposing Factor for Microbial Dysregulation

Following viral infection, the host responds to primary epithelial damage initiates or intensifies a eosinophilic inflammatory response in the nasal and sinus mucosa, leading to a shift in the residential microbiota. While upper respiratory tract infections are typically self-resolving, many patients with CRS subjectively relay that their initiating symptoms began with an upper respiratory infection (URI) that progressed in severity and chronicity [45,46].

Viruses are the most common causative agents of URI; the most frequent of these agents are the human rhinovirus, respiratory syncytial virus, influenza virus, parainfluenza virus, and coronavirus [45,46,47]. Of these pathogens, rhinovirus infection of lavage and mucosal samples was significantly higher in CRS patients than non-CRS controls, and parainfluenza virus was the second-most prevalent [46,47]. In a study in which healthy participants were inoculated with rhinovirus, the abundance of *Haemophilus influenzae*, *Neisseria subflavia*, and *Staphylococcus aureus* increased [3]. The mucosal inflammation and immune response triggered by these viruses may provide the disharmony needed for bacterial dysbiosis to develop.

Many patients presenting with acute viral infection also inappropriately receive an antibiotic prescription from their provider, contributing to antibiotic resistance and avoidable adverse events [48,49]. The etiological role of viruses and bacterial coinfection in the development of CRS should be explored.

## 8. Localized Persistent Immune Activation in Response to Inverted Papillomas

An inverted papilloma is a mucosal polypoid lesion prone to recurrence, which must often be histopathologically differentiated from polyps.

Tertiary lymphoid organs (TLOs) are aggregates of immune cells found in non-lymphoid tissues in response to inflammatory stimuli, such as microbes or pathogens. While helping hasten the clearance of acute infection, this enhanced immune response may exacerbate chronic inflammatory conditions such as CRS. TLO presence can be considered a localized response to persistent antigens.

Investigating TLO presence and pathological influence in nasal inverted papilloma (NIP) tissues, Bao et al. found a high prevalence of TLOs in NIP patients, while none were found in the nasal mucosa of healthy controls. TLO constituents were identified by cell surface markers, and found to be mainly composed of T cells, B cells, follicular dendritic cells, macrophages, and natural killer cells. TLOs were significantly associated with tissue eosinophilic infiltration and elevated local Th2 and Th17 expression compared to tissues without TLOs. NIP tissue containing TLOs had upregulated immunoglobulin production by TLO-resident B cells; IgE antibody levels were particularly higher compared to tissues without TLOs [50]. Perpetual immunological activation may contribute to inverted papilloma’s recurrence and chronicity. Nasal polyps in the environment of CRS may likewise be impacted by an unhindered localized immune response.

## 9. YAP-Associated Proliferation, Differentiation, and Neutrophil Infiltration in Patients with Nasal Inverted Papilloma

Nasal inverted papilloma (NIP) is often misdiagnosed as nasal polyps due to similar clinical features, but NIPs have a much higher rate of proliferation, which contributes to recurrence and malignant transformation. Yuan et al. analyzed the association with NIP and Yes-associated protein (YAP)—a transcriptional coactivator involved in regulation of cell proliferation, differentiation, and the apoptosis of airway epithelium [51].

YAP is a component of the Hippo pathway, a highly conserved signaling pathway which controls organ size by regulating cell proliferation and apoptosis, as well as regulating leukocyte expression in inflammation [52,53].

Yuan et al. investigated YAP’s influence on proliferation and inflammation within NIP pathogenesis [51]. Control patients (inferior turbinate, IT), patients with nasal polyps (NP), and patients with NIP (NIP) were compared; NIP patients were further categorized into three groups: Grade I, defined as ciliated respiratory epithelium with underlying squamous metaplasia; Grade II, partially ciliated epithelium with luminal squamous metaplasia and increased prominence of inversion; and Grade III, near-total absence of respiratory epithelium with dominant stratified squamous epithelium. YAP expression was analyzed in all groups by immunofluorescence (IF); proliferation was analyzed via Ki-67. YAP was found to be expressed significantly more in the NIP group than the NP and control group; YAP expression in both mRNA and protein levels increased with grade, with Grade III levels significantly higher. Further, a higher level of epithelial remodeling was associated with higher YAP-induced proliferation, leading to a reduction of both ciliated cells and goblet cells, suggesting the involvement of YAP in ciliogenesis. Increased YAP protein also significantly correlated with greater neutrophil infiltration [51]. More understanding of YAP’s involvement in inflammation and epithelial remodeling is needed.

## 10. Association of HPV and FoxM1 in Sinonasal Inverted Papillomas with Dysplastic Changes

Although sinonasal inverted papillomas (SIP) are benign, inward-growing neoplasms, they are known for invasiveness, recurrence, and a tendency towards malignant transformations [54]. SIP primarily affects the paranasal sinuses and nasal cavity. As our review found no terminological distinction between SIP and nasal inverted papilloma (NIP), this paper uses the terms interchangeably.

Malignant transformation of SIP has been reported in as many as 5–15% of all cases, but the mechanisms of malignant transformation remain incompletely understood. Epidermal growth factor (EGFR) mutations appear to be associated with SIP; as many as 88% of SIP were reported to exist with EGFR mutation [55]. Though HPV contributes to tumor formation by disrupting cellular proliferation and death, and has been found to upregulate EGFR expression, the association between HPV and SIP remains ambiguous. FoxM1, a transcription factor implicated in a number of malignancies, was identified as a biomarker involved in malignant transformation in SIP to sinonasal squamous cell carcinoma [54].

Rachmadi and colleagues described the association of EGFR mutation, HPV status, and FoxM1 expression with dysplastic changes in SIP [55]. The study divided 34 subjects with histologically-confirmed SIP diagnosis into one of two groups: 20 SIP samples with mainly inverted growth in epithelia without recognizable dysplasia and 14 SIP samples with dysplastic changes, without stromal invasion.

All samples of SIP with dysplasia were positive with FoxM1 immunohistochemistry, with a significant difference in FoxM1 expression between groups. HPV DNA was found in 25% of non-dysplastic SIP samples, versus 64.3% of SIP samples with dysplasia. EGFR mutations were present in 30% of the non-dysplastic group and 35.7% of the dysplastic group. Rachmandi and colleagues sobserved that HPV infection was more frequently detected in SIP with dysplasia rather than the non-dysplastic SIP group (*p* = 0.22). In 11 of the 24 patients with EGFR mutations, 6 in SIP group and 5 in SIP with dysplasia, there was no significant difference between the groups. FoxM1 expression was overexpressed in the SIP dysplasia group versus the SIP group (100% versus 10%, respectively; *p* < 0.001). FoxM1’s expression was also higher in HPV-positive samples (*p* = 0.022). There was a significant difference in HPV status and FoxM1 expression in non-dysplastic and dysplastic SIP (*p* = 0.022 and *p* < 0.001, respectively). No difference was found in EGFR mutation between groups [55].

**Figure 2:**
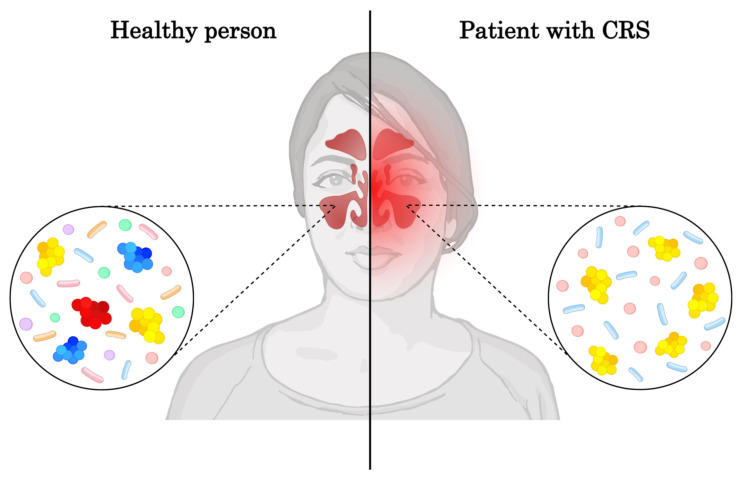
In the sinuses of healthy individuals, *Staphylococcus* spp., *Corynebacterium* spp., *Propionibacterium* spp., and *Achromobacter* spp. are dominant [13,19,27]. The most common and abundant species are *Staphylococcus epidermidis*, *Staphylococcus aureus*, and *Propionibacterium acnes*. Opportunistic bacteria, such as *Streptococcus pneumoniae*, *Neisseria meningiditis*, *Haemophilus influenzae* and *Moraxella catarrhalis*, occur less abundantly [13]. In the sinuses of CRSwNP patients, the most prevalent are *Staphylococcus* spp., *Lactobacillus* spp., and *Corynebacterium* spp. [27]. Although the amount of bacteria in the sinuses of healthy individuals is similar to that of CRSwNP patients, significant reduction of diversity of bacterial species was observed when compared to the sinuses of healthy individuals [40]. Figure created on NIAID Visual & Medical Arts. 11/15/2024. CRS. NIAID BIOART Source. bioart.niaid.nih.gov/bioart.

Due to the heterogeneity in how chronic rhinosinusitis (CRS) can manifest, the scope of this manuscript’s discussion was intentionally limited to the most relevant topics related to the sinonasal microbiome in a diseased state (Figure 2). Limitations thus arise within the manuscript from exclusion of certain topics, notwithstanding their importance, pertaining to CRS. Allergic fungal rhinosinusitis (AFRS) is not discussed in this paper, nor is immunocompromization and its relation to the AFRS endotype; nor is the effect of immunocompromization on general CRS presentation and therapy response. Discussion of hypersensitivity beyond eosinophilia is also limited, with absent mention of local allergic rhinitis. Acute rhinosinusitis (ARS), recurrent ARS, and disease development into CRS are also not discussed in this paper.

## 11. Conclusions

Chronic rhinosinusitis is a common inflammatory state of the paranasal sinuses. The role of the microbiome in CRS has yet to be clearly defined, but the multifactorial nature of the disease’s pathology is becoming increasingly revealed. Small cohort sizes and variability of sampling methods warrant study replication. Future investigation should involve further sinonasal microbial sampling among CRS patients and non-CRS controls to better define the populations of a pathogenic and non-pathogenic environment, respectively, and to determine how disease chronicity allows these residents to fluctuate in number.

The inflammatory response differs across a geographical spectrum, with eosinophil-dominant states more common in patients of Western countries. The host’s eosinophilic inflammatory response against acute viral infection may shift the resident sinonasal microbe population, predisposing to a dysregulated macrobiotic state.

Inverted papillomas are persistent, aggressive lesions that appear to arise from some transcriptional abnormalities, ultimately disrupting cell proliferation and apoptosis. Overexpression of certain biomarkers, namely FoxM1, is of interest in respect to their involvement in the malignant transformation of sinonasal inverted papilloma to sinonasal squamous cell carcinoma.

The CRS microbiome is characterized by a loss of diversity compared to the healthy population. While CRS and non-CRS patients may have the same bacterial load, CRS patients demonstrate significantly less diversity in their microbial population. *Corynebacterium* and *Staphylococcus aureus*, both present in healthy sinuses, increase and decrease in the CRS state, suggesting competition between these typical commensals. The abundance of *Campylobacter*, *Bdellovibrio*, and *Aggregatibacter* appears to increase in patients with recurrent nasal polyp growth.

Iatrogenic manipulation—such as long-term antibiotic and steroid use and surgeries altering the nasal landscape—may have a hand in the onset of dysbiosis. Disruption of the stable nasal microbiome may influence the severity and chronicity of sinusitis, and the extent of microbial interaction in the diseased state stands to be determined.

## Data Availability

Data sharing not applicable.

## Abbreviations

CRS—chronic rhinosinusitis; CRSwNP—CRS with nasal polyps; CRSsNP—CRS without nasal polyps; EPOS 2020—The European Position Paper of Sinusitis and Nasal Polyps 2020; AERD—aspirin exacerbated respiratory disease; NERD—NSAID exacerbated respiratory disease; COX1—cyclooxygenase 1; SIP—m sinonasal inverted papillomas; NIP—nasal inverted papilloma; EGFR—epidermal growth factor; HPV—human papilloma virus; AFRS—allergic fungal rhinosinusitis; ARS—acute rhinosinusitis.
